# An expression map for *Anopheles gambiae*

**DOI:** 10.1186/1471-2164-12-620

**Published:** 2011-12-20

**Authors:** Robert M MacCallum, Seth N Redmond, George K Christophides

**Affiliations:** 1 Division of Cell and Molecular Biology, Department of Life Sciences, Imperial College London, London, UK; 2 Pasteur Institute, 28 Rue Du Docteur Roux, Paris, 75015, France

## Abstract

**Background:**

Quantitative transcriptome data for the malaria-transmitting mosquito *Anopheles gambiae *covers a broad range of biological and experimental conditions, including development, blood feeding and infection. Web-based summaries of differential expression for individual genes with respect to these conditions are a useful tool for the biologist, but they lack the context that a visualisation of *all *genes with respect to *all *conditions would give. For most organisms, including *A. gambiae*, such a systems-level view of gene expression is not yet available.

**Results:**

We have clustered microarray-based gene-averaged expression values, available from VectorBase, for 10194 genes over 93 experimental conditions using a self-organizing map. Map regions corresponding to known biological events, such as egg production, are revealed. Many individual gene clusters (nodes) on the map are highly enriched in biological and molecular functions, such as protein synthesis, protein degradation and DNA replication. Gene families, such as odorant binding proteins, can be classified into distinct functional groups based on their expression and evolutionary history. Immunity-related genes are non-randomly distributed in several distinct regions on the map, and are generally distant from genes with house-keeping roles. Each immunity-rich region appears to represent a distinct biological context for pathogen recognition and clearance (e.g. the humoral and gut epithelial responses). Several immunity gene families, such as peptidoglycan recognition proteins (PGRPs) and defensins, appear to be specialised for these distinct roles, while three genes with physically interacting protein products (LRIM1/APL1C/TEP1) are found in close proximity.

**Conclusions:**

The map provides the first genome-scale, multi-experiment overview of gene expression in *A. gambiae *and should also be useful at the gene-level for investigating potential interactions. A web interface is available through the VectorBase website http://www.vectorbase.org/. It is regularly updated as new experimental data becomes available.

## Background

Genome sequencing [1] and gene expression microarray technologies have, in recent years, enabled systems-level research into the malaria-transmitting mosquito *Anopheles gambiae*. By measuring transcript levels with respect to biological events, such as blood feeding, development, parasite infection and mating, one can identify genes that are likely to be involved in the underlying processes. However, due to the wealth of information produced by individual experiments and the numerous leads that require further investigation, it is understandable that research groups rarely perform so-called meta-analysis of gene expression data, whereby multiple experiments are analysed simultaneously. Furthermore, meta-analysis is impeded by incompatibilities between different versions of genome annotations, microarray technologies, file formats, experimental designs, data processing pipelines and statistical analyses. Several ongoing projects are aiming to eliminate these inconsistencies and produce uniform processed and analysed data for the end user. Human curators at the two major microarray repositories, NCBI GEO [2] and Array Express [3], are working to produce enriched resources known as GEO Datasets and the Gene Expression Atlas [4], respectively. The VectorBase consortium [5] produces a similar unified gene expression resource for the invertebrate vector community.

Web-based expression summaries provide useful and concise biological overviews for individual genes of interest, however a common requirement is to know which other genes are expressed in a similar manner to a particular gene. GEO and ArrayExpress' curated expression resources provide such "nearest neighbour" gene lists, but within a single experiment only, not across multiple experiments. Some years ago, gene expression data from 553 *Caenorhabditis elegans *two-colour microarray experiments was clustered simultaneously to produce a 2D map known as TopoMap [6]. It was found that TopoMap clustered many genes of similar function, such as lipid metabolism, heat shock and neuronal genes. TopoMap is integrated into the WormBase genomics resource, but the underlying expression data is not available, reducing its utility. To the best of our knowledge, no large-scale meta-analysis of expression data has been made public for any other species.

Here we present a simple method for clustering expression data from a diverse set of microarray experiments. We have used data from *A. gambiae*, but the method is applicable to any organism. The results are visualised on a 2D map, and we show that many regions of the map are strongly linked to biological function. Two case studies are presented. One focuses on odorant binding proteins, which can be classified into several functional groups. The second looks at a large number of immunity-related genes, and likewise suggests specialised roles for members of several immunity gene families.

## Results and Discussion

### A map of *A. gambiae *gene expression

**Figure 1:**
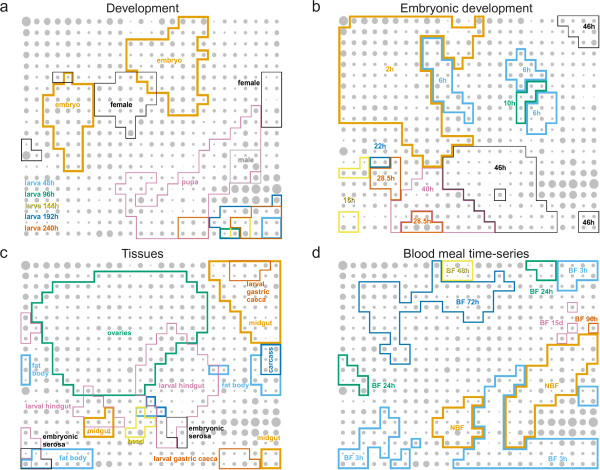
**The *A. gambiae *expression map**. Expression data from all publicly available experiments, representing 93 assay conditions, was summarised for 10194 genes and clustered using the self-organizing map algorithm. The area of the grey circles represents the number of genes mapping to each discrete node on the map (minimum 1, maximum 114). The four panels show the same map with different annotations that indicate regions of the map associated with high expression in various conditions: **a **Development from embryo to adult male and female [8,10]; **b **Embryonic development [17]; **c **Organs and tissues [8,10,11,17]; **d **Uninfected blood meal time course [8] (BF: blood fed, NBF: non-blood fed).

Using the VectorBase gene expression resource (1.0.7, June 2009) [5], gene-averaged expression values were extracted for 93 experimental conditions (see Table 1) derived from 11 publications [7-17]. After median-shift normalisation (see Methods for details), 10194 *A. gambiae *genes were clustered according to their expression data into a 25×20 grid of discrete clusters using the self-organizing map algorithm [18] with a Pearson correlation coefficient-based distance measure (see Methods for further details). The self-organizing map is randomly initialised; its iterative "training" or clustering algorithm is somewhat related to the *k*-means clustering method. However, unlike *k*-means, the 500 clusters on the self-organizing map are laid out in a meaningful order (two nearby clusters will usually have similar characteristics), although note that the "X" and "Y" axes have no predetermined meaning. Figure 1 illustrates how the high-dimensional expression data has been flattened into a two-dimensional grid, as a result of the competitive learning process. Gene expression space is highly convoluted, as indicated by the multiple discrete areas of high expression for many conditions (e.g. adult female, embryo 6 h, fat body, 3 h post blood meal).

**Table 1:** Experimental conditions

Experiment	condition(s)
Developmental series [10]	embryo:12-14 hours, larva:48 hours, larva:96 hours, larva:144 hours, larva:192 hours, larva:240 hours, pupa:240 hours, adult:312 hours
Adult female tissues [10]	head, midgut, ovaries, carcass
Odumasy vs. Kisumu strain [9]	Odumasy v Kisumu
Blood meal time series [8]	Non-blood-fed, Blood-fed 3 h, Blood-fed 24 h, Blood-fed 48 h, Blood-fed 72 h, Blood-fed 96 h, Blood-fed 15 d
Blood-fed adult female tissues [8]	midgut, fat body, ovaries
Alimentary canal compartments [11]	gastric caeca, anterior midgut, posterior midgut, hindgut, whole organism
Larval salivary glands [16]	salivary gland, whole organism
Blood meal after 15 days [8]	Non-blood-fed 18 d, Blood-fed 15 d
Male vs. female [8]	male, female
Two consecutive blood meals [8]	Non-blood-fed, Blood-fed 24 h, Blood-fed twice
Larval and adult stages [8]	larva, adult
Male vs. female [10]	male, female
Plasmodium berghei midgut invasion time-series [7]	wild-type parasite infection v invasion-deficient parasite infection:before midgut invasion, wild-type parasite infection v invasion-deficient parasite infection:during midgut invasion, wild-type parasite infection v invasion-deficient parasite infection:after midgut invasion
Plasmodium berghei midgut invasion stage comparisons [7]	wild-type parasite infection:during midgut invasion v before midgut invasion, invasion-deficient parasite infection:during midgut invasion v before midgut invasion, wild-type parasite infection:after midgut invasion v during midgut invasion, invasion-deficient parasite infection:after midgut invasion v during midgut invasion
M and S form 4th instar larvae [15]	M form, S form, M form:M-GA-CAM, M form:Mali-NIH, S form:KIST, S form:Pimperena
M and S form virgin females [15]	M form, S form, M form:M-GA-CAM, M form:Mali-NIH, S form:KIST, S form:Pimperena
M and S form gravid females [15]	M form, S form, M form:M-GA-CAM, M form:Mali-NIH, S form:KIST, S form:Pimperena
Permethrin-resistant strain [12]	permethrin-selected v unselected
Mated females [13]	virgin 0 h, mated 2 h, mated 6 h, mated 24 h
Chloroquine exposure [14]	chloroquine v none:Plasmodium berghei infected, chloroquine v none:uninfected
Embryonic development [17]	2 h, 4 h, 6 h, 7 h, 8 h, 10 h, 13 h, 16 h, 19 h, 22 h, 25 h, 28.5 h, 31 h, 34 h, 37 h, 40 h, 43 h, 46 h
Embryonic serosa [17]	embryonic serosa, embryo

The 93 experimental conditions used in the VectorBase release 1.0.7 expression map are listed.

Given the assumed difficulty of mapping such high-dimensional data into two dimensions, how reproducible are the maps with respect to the random initialisation step? A simulation, based on an additional 100 randomly seeded maps (not shown), was performed to see how often genes that are co-clustered in the "main" map (shown in the figures) would co-cluster in a re-mapping. It was found that 9907 of 50,000 (20%) randomly selected co-clustered gene pairs co-cluster again in a randomly selected re-mapping, while 40,747 (81%) of gene pairs re-map to the same or "nearby" clusters (≤ 5 grid units separation). This indicates that the general topology of the map is reproducible, although the fine details may not always be.

### Map nodes and regions are enriched with respect to gene function

**Figure 2:**
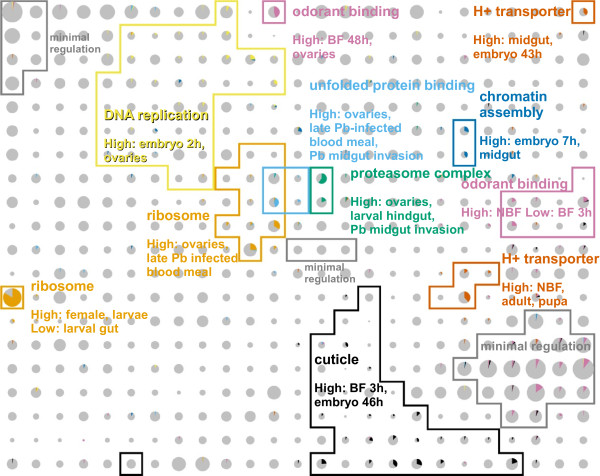
**Regions of the map are enriched with different gene functions**. Genes annotated with a selection of (highly enriched on a per-node basis) Gene Ontology terms are indicated by the coloured pie charts in each node. The corresponding regions marked with coloured lines are described according to their gene expression characteristics. The Gene Ontology terms are as follows: orange, GO:0003735, structural constituent of ribosome; light blue, GO:0051082, unfolded protein binding; green, GO:0000502, proteasome complex; yellow, GO:0006260, DNA replication; dark blue, GO:0031497, chromatin assembly; vermillion, GO:0015078, hydrogen ion transmembrane transporter activity; purple, GO:0005549, odorant binding; black, GO:0042302, structural constituent of cuticle.

The gene sets corresponding to each map node were tested for enrichment in annotated function through a Gene Ontology (GO [19]) term over-representation analysis [20]. A large number of biological processes, molecular functions and cellular components were found to be enriched. Genes annotated with a small selection of these GO terms are highlighted in Figure 2, where the coloured pie slices within the grey circles indicate the proportion of genes with these GO terms. Components of macromolecular complexes, such as the ribosome and proteasome are among the most highly enriched terms, which is expected since these proteins need to be produced in stoichiometric amounts and are therefore likely to be coregulated. Non-complex associated genes are also highly clustered by the map, such as those involved in polysaccharide metabolism and odorant binding. A full list of highly significant GO terms is provided in Table 2.

**Table 2:** Over-represented Gene Ontology terms *P <*1 × 10^-6^

P	Accession	Type	Description
0	GO:0003735	MF	structural constituent of ribosome
0	GO:0005840	CC	Ribosome
0	GO:0006412	BP	Translation
8.37899e-40	GO:0000502	CC	proteasome complex
2.895e-25	GO:0004298	MF	threonine-type endopeptidase activity
6.398e-25	GO:0015077	MF	monovalent inorganic cation transmembrane transporter activity
7.681e-24	GO:0005811	CC	lipid particle
8.87e-24	GO:0030163	BP	protein catabolic process
1.163e-19	GO:0005549	MF	odorant binding
1.358e-19	GO:0005829	CC	Cytosol
5.049e-19	GO:0051082	MF	unfolded protein binding
2.496e-18	GO:0043632	BP	modification-dependent macromolecule catabolic process
2.496e-18	GO:0034962	BP	cellular biopolymer catabolic process
1.657e-16	GO:0004888	MF	transmembrane receptor activity
2.507e-16	GO:0006457	BP	protein folding
2.709e-16	GO:0006508	BP	Proteolysis
1.096e-15	GO:0007586	BP	Digestion
1.178e-14	GO:0019829	MF	cation-transporting ATPase activity
1.236e-14	GO:0009201	BP	ribonucleoside triphosphate biosynthetic process
1.236e-14	GO:0009205	BP	purine ribonucleoside triphosphate metabolic process
1.236e-14	GO:0009145	BP	purine nucleoside triphosphate biosynthetic process
1.245e-14	GO:0055085	BP	transmembrane transport
1.245e-14	GO:0016469	CC	proton-transporting two-sector ATPase complex
1.788e-14	GO:0006818	BP	hydrogen transport
2.249e-14	GO:0016651	MF	oxidoreductase activity, acting on NADH or NADPH
4.651e-14	GO:0006119	BP	oxidative phosphorylation
8.567e-14	GO:0009152	BP	purine ribonucleotide biosynthetic process
2.569e-13	GO:0016675	MF	oxidoreductase activity, acting on heme group of donors
2.569e-13	GO:0015002	MF	heme-copper terminal oxidase activity
3.205e-12	GO:0007606	BP	sensory perception of chemical stimulus
1.248e-11	GO:0005783	CC	endoplasmic reticulum
5.56e-11	GO:0000786	CC	Nucleosome
6.352e-11	GO:0031497	BP	chromatin assembly
1.354e-10	GO:0034728	BP	nucleosome organization
1.421e-10	GO:0015672	BP	monovalent inorganic cation transport
3.172e-10	GO:0042302	MF	structural constituent of cuticle
9.588e-10	GO:0009109	BP	coenzyme catabolic process
9.936e-10	GO:0006084	BP	acetyl-CoA metabolic process
1.003e-09	GO:0065004	BP	protein-DNA complex assembly
1.181e-09	GO:0009060	BP	aerobic respiration
4.353e-09	GO:0004252	MF	serine-type endopeptidase activity
5.505e-09	GO:0033554	BP	cellular response to stress
1.191e-08	GO:0006040	BP	amino sugar metabolic process
1.359e-08	GO:0005976	BP	polysaccharide metabolic process
2.448e-08	GO:0006260	BP	DNA replication
7.826e-08	GO:0006974	BP	response to DNA damage stimulus
8.827e-08	GO:0008061	MF	chitin binding
1.51e-07	GO:0005739	CC	Mitochondrion
2.35e-07	GO:0045184	BP	establishment of protein localization
2.664e-07	GO:0034613	BP	cellular protein localization
4.102e-07	GO:0031326	BP	regulation of cellular biosynthetic process
6.422e-07	GO:0005344	MF	oxygen transporter activity
6.535e-07	GO:0003676	MF	nucleic acid binding
6.985e-07	GO:0010556	BP	regulation of macromolecule biosynthetic process
7.582e-07	GO:0019219	BP	regulation of nucleobase, nucleoside, nucleotide and nucleic acid metabolic process

A list of GO terms found to be over-represented in the genes of at least one of the 500 clusters on the map. Only GO terms associated with four or more genes per cluster and with a corrected *P*-value less than 1 × 10^-6 ^are shown. Child and parent terms with higher *P*-values than those shown are omitted for clarity. BP = biological process, MF = molecular function, CC = cellular component.

Highly enriched gene functions are frequently found in multiple distinct regions of the map, indicating major differences in their expression and hence the biological context in which the genes operate. Some examples are discussed below.

The strongest enrichment of function is seen for the ribosomal proteins on the left hand edge of the map. However, there is a second group of ribosomal protein genes in the centre of the map that is characterised by high expression in ovaries and is therefore likely to be involved in egg production.

Genes involved in DNA, RNA and protein synthesis are generally found above the diagonal from lower-left to upper-right. Temporally, spatially or functionally related metabolic functions are often co-located on the map. For example, near the centre of the map, clusters enriched in protein synthesis (ribosome), protein folding and protein degradation (proteasome) are found together. Additional file 1, Figure S1 shows a wider selection of DNA/RNA/protein metabolic functions and their relationships (e.g. the proximity of DNA replication and repair, transcription and RNA processing, and protein synthesis and protein transport).

One additional gene function analysis was performed. The null hypothesis asserts that the genes annotated with a particular GO term are randomly distributed across the map, and this is tested empirically. Table 3 lists the 59 GO terms for which this null hypothesis is rejected at *P <*0.01 (after multiple testing correction), that is, functions which are non-randomly distributed on the map.

**Table 3:** Non-randomly distributed Gene Ontology terms *P <*0.01

GO accession	# Genes	GO description
GO:0003677	339	DNA binding
GO:0006355	238	regulation of transcription, DNA-dependent
GO:0006118	229	electron transport
GO:0004252	223	serine-type endopeptidase activity
GO:0003700	167	transcription factor activity
GO:0006412	157	translation
GO:0004872	138	receptor activity
GO:0045449	137	regulation of transcription
GO:0005549	126	odorant binding
GO:0003735	117	structural constituent of ribosome
GO:0003723	116	RNA binding
GO:0004871	114	signal transducer activity
GO:0005840	113	Ribosome
GO:0008233	102	peptidase activity
GO:0006811	100	ion transport
GO:0007242	94	intracellular signaling cascade
GO:0004930	94	G-protein coupled receptor activity
GO:0007186	93	G-protein coupled receptor protein signaling pathway
GO:0042302	80	structural constituent of cuticle
GO:0005216	77	ion channel activity
GO:0001584	77	rhodopsin-like receptor activity
GO:0030529	75	ribonucleoprotein complex
GO:0006350	73	transcription
GO:0007608	71	sensory perception of smell
GO:0004984	70	olfactory receptor activity
GO:0004386	70	helicase activity
GO:0006886	64	intracellular protein transport
GO:0007476	55	imaginal disc-derived wing morphogenesis
GO:0030528	52	transcription regulator activity
GO:0008061	49	chitin binding
GO:0006030	49	chitin metabolic process
GO:0048477	48	Oogenesis
GO:0008026	48	ATP-dependent helicase activity
GO:0007018	39	microtubule-based movement
GO:0006814	39	sodium ion transport
GO:0007517	38	muscle development
GO:0006334	34	nucleosome assembly
GO:0005694	34	chromosome
GO:0003777	34	microtubule motor activity
GO:0007049	33	cell cycle
GO:0006396	32	RNA processing
GO:0006281	31	DNA repair
GO:0003774	29	motor activity
GO:0000786	29	nucleosome
GO:0007156	28	homophilic cell adhesion
GO:0048749	27	compound eye development
GO:0005198	27	structural molecule activity
GO:0015986	26	ATP synthesis coupled proton transport
GO:0004175	26	endopeptidase activity
GO:0046961	25	proton-transporting ATPase activity, rotational mechanism
GO:0046933	25	hydrogen ion transporting ATP synthase activity, rotational mechanism
GO:0043234	25	protein complex
GO:0016469	25	proton-transporting two-sector ATPase complex
GO:0006260	24	DNA replication
GO:0003899	23	DNA-directed RNA polymerase activity
GO:0001745	23	compound eye morphogenesis
GO:0000785	23	chromatin
GO:0006461	22	protein complex assembly
GO:0005643	22	nuclear pore

A list of GO terms for which the genes annotated with them are found to be non-randomly distributed on the map. Only GO terms associated with between 20 and 400 genes were analysed. The test for non-random distribution and multiple testing correction is described in the Methods section.

### Multi- *vs*. single-experiment maps

**Figure 3:**
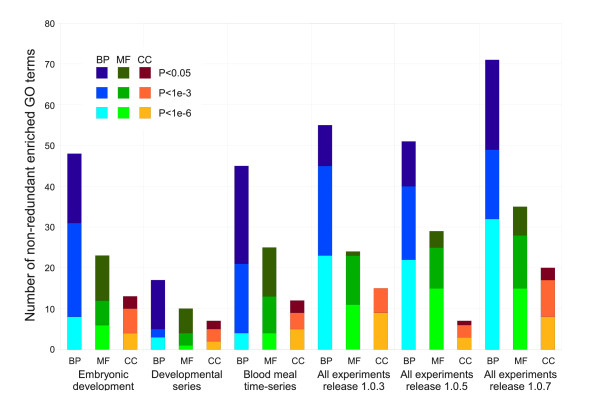
**Multi-experiment maps cluster genes by function to a greater extent than single experiment maps**. The number of enriched non-redundant Gene Ontology terms is shown for three significance thresholds for six different maps: three single experiment maps, two maps made with older versions of the VectorBase expression data, and the map using current data as shown in previous figures. The multi-experiment maps show substantially more clustering of genes by biological process and molecular function than the single experiment maps. Full details of the maps and datasets can be found in Table 4.

Some of the experiments included in this analysis, for example the life-cycle series [10], blood meal time series [8] and embryonic developmental series [17], have a large enough number of experimental conditions to allow effective clustering of genes on their own. Using an identical self-organizing map approach on data from these individual experiments, we found significant enrichment of gene function in the resulting clusters, as shown in Figure 3. However the number of enriched GO terms (reflecting the breadth of biology visualised in the map) was lower than that obtained with the all-experiment (VectorBase release 1.0.7) map. The difference is particularly clear at the *P <*1 × 10^-6 ^threshold.

The mapping was also performed with two older releases of the expression data (release 1.0.3, January 2009 and release 1.0.5, July 2008), which contain fewer experiments (see Table 4). In general there is an increase in the number of enriched GO terms as new experiments are added, although there is a small drop in the number of biological process terms from release 1.0.3 to 1.0.5. From this limited data we tentatively predict that the incorporation of future experimental data will produce increasingly informative maps. Further discussion on the inclusion/exclusion of experiments can be found below (see section **Limitations**).

**Table 4:** Map information

Map name	# Conditions	Publications	# Genes	Map dimensions
Embryonic development	17	[17]	9959	25×20
Developmental series	8	[10]	3125	15×10
Blood meal time-series	7	[8]	9959	25×20

All experiments release 1.0.3	46	[7-11]	10194	25×20
All experiments release 1.0.5	71	+ [12-15]	10194	25×20
All experiments release 1.0.7	93	+ [16, 17]	10194	25×20

Details of the number of experimental conditions, publications (as citations), number of mapped genes (genes with experimental data) and map size are provided as a supplement to Figure 3.

### Case study: odorant binding proteins

Odorant binding proteins (OBPs [21]), which transport odorant molecules through the extracellular fluid of chemosensilla to transmembrane odorant receptor (OR) proteins on olfactory receptor neurons, are found in three main regions of the expression map (Figure 2, two in purple and one in grey labelled "minimal regulation", bottom right). One region is characterised by minimal differential regulation and presumably represents constitutively expressed genes (although we cannot rule out differential expression in some yet-to-be-performed experiment). Another region rich in OBPs is characterised by high expression in non-blood fed females, suggesting a role in mating or host seeking for these genes. The third cluster is expressed after blood feeding and may be implicated in locating suitable sites for egg laying. A similar functional hypothesis for OBPs, based on blood meal data alone, has already been proposed [8], however this only identified three of the most differentially expressed genes, whereas the expression map classifies the majority of this large family into three, or perhaps more (see below), functional groups. In contrast however, the vast majority of ORs (as defined by InterPro domain IPR004117) appear to be unregulated (data not shown, but easily available via the web interface).

**Figure 4:**
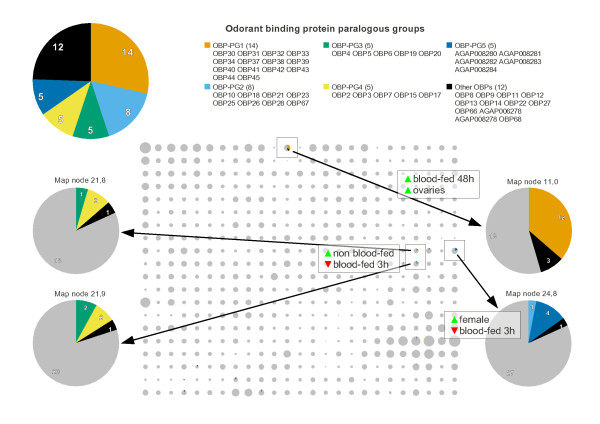
**Odorant binding proteins (OBPs) are found in several distinct regions of the expression map, which generally correspond to paralogous groups**. The OBP paralogous groups (OBP-PGs) are defined (top). OBPs are shown as coloured pie sections on the expression map (centre) with regions of interest are outlined and annotated in terms of two major expression characteristics. The pie charts of four map nodes dominated by OBPs are shown at greater magnification (bottom left and right).

We observe a large degree of overlap between OBP expression map clusters and paralogous groups (OBP-PGs) defined in the VectorBase comparative genomics database (Figure 4). This is to be expected since gene duplication events are likely to involve "upstream" regulatory DNA, and so transcripts of duplicated genes are likely to be co-regulated. However, paralogous OBPs are somewhat divergent (20- 25% pairwise identity at the protein level is common) so it is notable that their transcriptional regulation has been conserved. The phylogenetic analysis splits the "high in non-blood fed females" region (Figure 2, middle right) into two sub-regions (Figure 4): on the left, two clusters containing most members of OBP-PG3 and OBP-PG4, and on the right, a cluster dominated by OBP-PG5, which consists of a tandem array of five hitherto unannotated [21] OBPs (*AGAP008280-AGAP008284*) with no known orthologues in *Drosophila melanogaster *or other mosquitoes. *AGAP008280 *is more highly expressed in males while *AGAP008281-AGAP008284 *have very much higher expression in females. This combination of sex-specific expression and species-specific phylogeny suggests a role for OBP-PG5 genes in mating, perhaps supplementary to wing-beat frequency matching [22]. Given that members of OBP-PG3 and OBP-PG4 have *Drosophila *orthologues, these OBPs may be involved in sugar-based meal seeking rather than blood meal host seeking. OBP-PG1 members have only mosquito orthologues, which supports the suggestion above that these genes may be involved in oviposition site selection (the larval habitats of fruit flies and mosquitoes are very different).

### Case study: immunity genes

**Figure 5:**
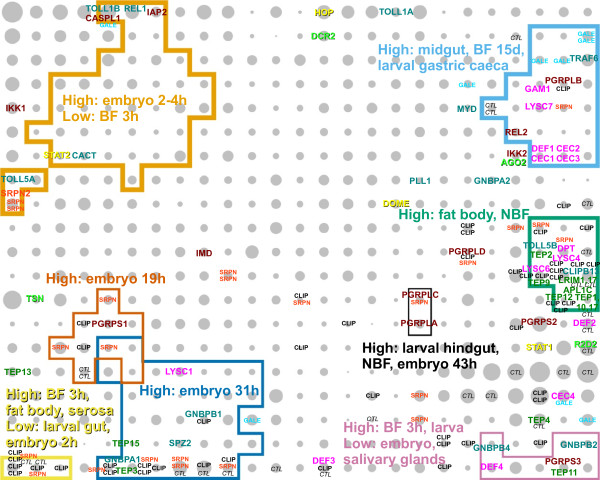
**Immunity genes are highly localised**. Genes belonging to various immunity-related pathways and gene families are shown on the map. Toll pathway members are labeled in dark cyan, the IMD pathway in dark red, JAK/STAT pathway in yellow, siRNA pathway in bright green, anti-microbial peptides and effectors in magenta, and LRIM1, APL1C and TEPs are labeled in dark green. CLIP-domain serine proteases, serpins, C-type lectins and galectins are shown generically in black, light red, black italic and cyan respectively. Several regions of the map that are rich in immunity genes are outlined and described by their dominant gene expression characteristics.

In Figure 5, immunity-related genes, including *Toll*, *IMD*, *JAK*/*STAT *and siRNA pathway members, and anti-microbial effectors such as defensins and cecropins are marked on the expression map. These genes show a very clear non-random distribution (P < 0.001), and occupy several distinct regions of the map. Genes from the four main immune pathways are generally intermingled, rather than in pathway-specific separate clusters. This possibly reflects the shared or similar biological contexts (e.g. timing and tissues) in which the various immune challenges are encountered and cleared. The intermingling may also suggest crosstalk between the pathways.

The genes *REL1 *and *REL2*, encoding the core transcription factors of the Toll and IMD pathways respectively, have very different expression profiles. This is perhaps expected since *REL1*, an orthologue of *Drosophila dorsal*, and other *Toll *pathway members have well documented roles in dorso-ventral pattern formation in the early embryo, and indeed we see *TOLL1B*, *TOLL5A*, *REL1*, and *CACT *in the early embryo region of the map. Notably, *TOLL1A, 1B, 5A *and *5B *are co-orthologues of *Drosophila Toll*, which codes for a transmembrane receptor with developmental and immune roles. One can speculate that, of these four mosquito receptors, *TOLL1B *is the most likely functional orthologue of *Toll *as it clusters closely with *REL1 *on the map. However, the location of *TOLL5B *close to many other immunity genes (Region labelled "High: fat body, NBF", middle right) as well as that of *TOLL5A *close to *TOLL1B*, *REL1 *and *CACT *may imply that at least three of the four co-orthologues of *Toll *play central, but likely distinct, roles in mosquito immunity.

Many of the major immunity gene family members are quite widely dispersed on the map. For example, the anti-microbial cecropin genes *CEC1*, *CEC2 *and *CEC3 *are tightly clustered in a region characterised by strong midgut expression and low expression 3 h post blood meal (top right), while *CEC4 *is located quite far away in a region with less overall differential expression and a mild positive response at 3 h post blood meal (lower right). This suggests that cecropins 1-3 have similar roles but are perhaps specialised to counter a range of pathogens, while *CEC4 *has evolved to perform a different role. The four defensins have a similarly informative distribution: *DEF1 *is with the main cluster of cecropins suggesting it has a similar function, while the others are in the lower right corner where the 3 h post blood meal response is strong. In particular, *DEF3 *is clustered with a large number of cuticle genes, suggesting a role in immunity during blood meal induced cuticle expansion, perhaps against fungal infection.

The peptidoglycan recognition proteins (PGRPs) are another gene family whose functional diversity is reflected in the map. All PGRPs, as their name implies, are able to bind microbial peptidoglycan specifically but some are believed to have catalytic activity due to the conservation of three active site amino acids [23-25]. In *A. gambiae*, the putative catalytic members of the family are *PGRPLB*, *PGRPS2 *and *PGRPS3*. Interestingly these three genes all map to the right-most edge of the map. *PGRPLB *lies in a region populated by other effector genes and peptides (*GAM1 *(*AGAP008645*), *LYSC7*, *DEF1 *and cecropins 1-3), supporting its proposed role as an antimicrobial agent. *PGRPS2 *and *PGRPS3 *map close to *DEF2 *and *DEF4 *respectively, suggesting parallel but as yet unidentified roles.

The recently described physical interactions between two leucine-rich repeat (LRR) proteins LRIM1 (AGAP006348) and APL1C and the complement C3-like protein TEP1 [26] are mirrored in the expression map; the two LRR genes map to the same grid node while TEP1 maps to the node below. These proteins are implicated with the activation of the mosquito complement system, with TEP1 being shown to localise around invading *Plasmodium berghei *ookinetes. This region of the map (Figure 5, green outline, middle right) has the highest density of immunity genes, including many other TEPs (1,2,9,10,12,17), CLIP-domain serine proteases, and one additional member of the recently characterised LRIM family [27], *LRIM17 *(*LRRD7*), which has been shown through RNAi mediated knockdowns to affect *Plasmodium *ookinete invasion [28].

### Limitations

Although the clustering of genes based on their expression in many different experiments appears to be successful--as assessed by the co-clustering of genes with similar function, at least--the methodology has some potential shortcomings which merit discussion.

Since data from so many experimental conditions is presented in one place there is the possibility that users could over-interpret map cluster expression summaries. For example, genes in cluster 22,9 could be (wrongly) interpreted as having "high expression in the fat bodies of non blood-fed females". However, the fat body assays used tissue from *blood-fed *females, so the correct summary should be "high expression in non blood-fed females and the fat bodies of blood-fed females". Users should be aware that very few of the possible combinations of experimental conditions have actually been assayed.

The use of different mosquito strains from one laboratory to another may also make interpretation of the map more difficult. First, polymorphisms may differentially alter microarray hybridisation efficiency in one strain relative to another for certain genes. However, this would appear to have a minimal confounding effect, since microarray studies have directly compared different strains and the results have been successfully validated with quantitative PCR [15,29]. Second, strains may actually exhibit biologically meaningful differences in expression (e.g. a gene may be highly expressed in the midgut of one strain but not in another). On first impressions this may seem like a problem, but it is actually an advantage because the differential (inter-experiment) expression resulting from strain differences (and other sample characteristics, such as sex, rearing conditions, etc) simply provides data with which finer-grained clustering can be obtained. The web interface, however, could be enhanced in future versions to display all available sample characteristics. Currently only the most pertinent information is available in the experiment titles (e.g. "Adult female tissues").

While we have re-analysed all data in order to standardise the statistical treatment there is still a possibility that technical differences between microarray technologies (platforms) could affect the meta-analysis. For example, platforms with a wider range of detection are capable of producing data with greater dynamic range. If high and low dynamic range datasets are mapped together, the high dynamic range data will have a greater influence on the clustering of genes. However, the dynamic range of expression data can also be influenced by the relative severity of the experimental conditions being tested (for example a 10°C heat shock will cause greater magnitude gene expression changes than a 1°C heat shock [30]). The VectorBase 1.0.7 expression data set contains both high and low dynamic range experiments (Additional file 2, Figure S2). The low dynamic range experiments tend to involve less severe conditions, such as strain comparisons. If datasets were range-normalised prior to mapping, the biological relevance of very highly regulated genes would be lost.

Another limitation is that we discard/ignore the statistics relating to the mean expression values used as input data to build the map. For instance, the numbers of replicates and standard deviations could be used to filter out bad data or to produce Gaussian models for each expression value (with which the map could be trained). Such enhancements, if implemented, would likely improve the quality of the mapping still further.

We have tried to keep the number of parameters in our approach to a minimum, however the size and shape of the map has a major effect on the outcome and was decided somewhat arbitrarily. In general, small maps produce large gene clusters, while large maps produce smaller clusters. For any given biological annotation, the extent of its enrichment *within *clusters will depend on cluster size and the number of genes annotated as such (i.e. in a large map, members of a large gene family may be spread across many neighbouring nodes but not be significantly enriched in any one node; while in a smaller map, significant enrichment in one large cluster may be seen). Thus, no map size is optimal in all cases. The dimensions of the VectorBase *A. gambiae *expression map (25 × 20) were chosen to give an average of 20 genes per cluster--a manageable number. Alternative map sizes could be provided by VectorBase in the future.

VectorBase strives to be unbiased and include all data for its core species in the expression database, particularly those with raw data deposited in public repositories. However, for technical reasons, total coverage of experiments cannot be guaranteed. Furthermore, in the mosquito field there is quite a heavy experimental bias (for example, the majority of data comes from female mosquitoes). As the VectorBase resource expands, questions arise as to what to do with largely redundant datasets (there are now three adult tissue experiments: [8,10,31]). Multiple assays of similar conditions or tissues (albeit with strain and rearing differences, see above) will proportionally shift the focus of the map towards those conditions or tissues; less space will be available for the allocation of genes into clusters based on other expression characteristics. One solution may be to perform some pruning of redundant datasets, another may be to produce specialist maps (e.g. developmental studies only) in addition to the "all conditions" map.

## Conclusions

One obvious use for the *A. gambiae *expression map is to short-list potential interaction partners for proteins of interest. For example, one can extrapolate from the recent findings for *LRIM1 *[26] that other LRIM family members will form heteromeric complexes and perhaps also interact with one or more TEPs, and that these genes will, like *LRIM1*, *APL1C *and *TEP1*, probably also be co-located on the map. Similarly, we observe a general tendency for CLIP-domain serine proteases and serpin family serine protease inhibitors (marked "CLIP" and "SRPN" in Figure 5 respectively) to be clustered together in many areas of the map, which suggests that the experimental elucidation of enzyme-inhibitor relationships can be greatly accelerated using the map.

A further advantage of performing clustering on all available data is that the clusters obtained are likely to be fine-grained enough for promoter analysis aimed at the discovery of *cis*-regulatory DNA sequences responsible for co-regulation. Post-transcriptional regulatory mechanisms (e.g. endogenous miRNA) will also be responsible for some of the observed co-regulation, so transcript-based signals (e.g. miRNA targets) might also be detectable in expression map clusters.

Finally, we propose a role for expression maps in comparative transcriptomics. Current approaches compare data from two or more broadly equivalent experiments that have been performed in two or more organisms (e.g. developmental stages in *A. gambiae *and *Drosophila melanogaster *[10]). If the experiments are performed in different laboratories and at different times, the experimental designs are likely to be different enough to invalidate or at the least complicate the analysis. However, expression maps tend to smooth out these differences, so that intra-map distances between pairs of orthologous genes should be robustly comparable between species, especially if the maps have been generated using a similar set of experiments. One can also quantify the functional divergence of gene families by measuring their intra-map dispersal, and compare these between species.

## Availability

The *A. gambiae *expression map has potential for making expression data more accessible and useful to researchers throughout the field. A web interface is available at http://funcgen.vectorbase.org/ExpressionMap/Anopheles_gambiae/paper -- showing the data presented in this paper. However, as the resource is updated as part of VectorBase's release cycle, newer versions are also available. While this manuscript was being revised an expression map for the Dengue vector *Aedes aegypti *was also made available. In addition, the source code for map generation and web visualisation is available under the GNU General Public License at https://github.com/VectorBase/ExpressionMap.

## Methods

### Data preparation

All data was obtained from the VectorBase gene expression resource, which is a curated collection of published, publicly available gene expression data for invertebrate vectors of human pathogens. The standard VectorBase curation pipeline begins with importing original raw data files, obtained from GEO [2], ArrayExpress [3] or the authors, into the microarray data management system BASE [32]. Low quality data is then removed according to the authors' quality flags. Intensity data is normalised with either the Lowess algorithm [33] for two colour data, or the RMA algorithm [34] for single channel data, using the relevant BASE plugin with default parameters. All ratio or intensity values for a given gene and hybridisation combination (there may be multiple reporters per gene and/or multiple spots per reporter) are summarised by their mean. The means from multiple hybridisations for the same experimental condition (these are usually biological replicates, or less often, technical "dye swap" replicates) are then averaged again to give a single value per gene and condition combination. The number of averaged data points and their variance are discarded (see Results and Discussion: "Limitations").

Some microarray technologies and experimental designs produce intensity values whose absolute values cannot always be compared directly from gene to gene. These include single channel technologies and some two colour experiments using global reference samples. With this kind of data, it is only possible to calculate correlation coefficients between gene expression profiles within a single experiment. Some form of normalisation is needed to give expression values from different "reference-less" experiments a common reference point so that multi-experiment expression profiles can be compared. We chose to apply a "median shift" normalisation step to such ratios and intensity values. In median shift normalisation, each expression profile is centred around zero by subtracting its median value (example: for a gene with expression values in one particular experiment (say, three tissues) being 11,4, and 6, the normalised values will be 5, -2, and 0). The median-shift normalised data for 10194 genes and 93 experimental conditions is available from the VectorBase download page.

### Self-organising map

The expression data was clustered using the self-organizing map algorithm as follows. Unless otherwise stated, the map dimensions were 25×20, the starting learning rate was 0.1, and the starting neighbourhood radius was 10. Prior to training, the map was randomly initialised with values within the range of the expression data. During the training of a self-organizing map, input vectors are compared with reference vectors at each map node (henceforth: "node vectors"). These vectors have the same number of dimensions as the input data (93 in this case). In this work, the comparison is made with the Pearson correlation coefficient, and missing values are simply excluded from the calculation. (The Euclidean distance measure was also tried and gave similar results.) The node vector with the highest correlation and its neighbours within a specified radius are updated towards the input vector by an amount proportional to the learning rate. As training proceeds, input vectors are "presented" to the map at random (with replacement) on average 20 times each while the learning rate and neighbourhood radius are linearly reduced towards zero. When training is complete, genes are assigned for a final time to their closest node. Each node vector can be thought of as a mean expression vector (or profile) for the genes mapping to that node. The algorithm attempts to preserve the topology of the high dimensional input data in the two-dimensional mapping, however the two axes of the map have no predetermined meaning.

The algorithm was implemented in Perl and PDL (Perl Data Language), and the maps are stored in a relational database through the object oriented Class::DBI interface. All source code is available under the GNU General Public License at https://github.com/VectorBase/ExpressionMap.

### Map outlines

The coloured outlines in Figures 1, 2 &5 indicate regions where one or more node vector components satisfy a simple arithmetic inequality. For example, the orange outlines marked "embryo" in Figure 1a highlight map nodes where the node vector component for embryo expression [10] is greater than 0.25. The choices of node vector component and thresholds was largely arbitrary, with an emphasis on simplicity and clear visualisation. For Figures 2 &5, nodes of interest (e.g. with a large fraction of genes with a particular function) were chosen manually and vector component thresholds were determined in a semi-automatic fashion. Different thresholds may be explored interactively via the web interface.

### Gene function over-representation analysis

The self-organizing map presented in Figures 1, 2 &5 consists of 500 nodes, each of which can be considered as a gene cluster. We applied a Gene Ontology (GO) over-representation analysis as implemented in the program ErmineJ [20] on each cluster. The analysis uses Fisher's Exact Test and the null hypothesis states that genes with a particular GO term are randomly distributed between the cluster of interest and the rest of the map. GO terms that are associated with less than ten or more than a quarter of the genes on the map were excluded from the analysis as they are generally not informative. The GO term database of 2009/03/02 was used to defined GO term relationships, and the GO annotations for *A. gambiae *genes were retrieved from VectorBase BioMart on the same date.

The *P *values reported from the GO analysis are corrected for multiple testing (*>*1000 GO terms are tested) according to the Benjamini-Hochberg false discovery rate (FDR) procedure, and correspond to the minimum FDRs (false positives as a fraction of all positives) at which the null hypotheses can be rejected. This correction does not take into account overlaps between parent and child GO terms.

Additionally, a GO term is only reported as enriched if four or more genes in the cluster are annotated with that term.

### Empirical non-random distribution test

The over-representation analysis described above is not ideal in situations where genes with a particular function are localised within the map, but are not necessarily confined to one map node/cluster. We therefore implemented a sampling-based test to quantify the general non-randomness of a gene set on the map as follows. For the set *N *of *n *genes of interest located on the map we calculate the mean, *d*, of the city block distance to their closest neighbours within *N*. Then, sets *N*' of *n *genes are randomly sampled from the map 100 times. For each sample of genes, their mean distance to closest neighbour *d*' is calculated as above and compared with the "true" value *d*. For a non-randomly distributed set of genes, *d*' is not likely to be smaller than *d*. The estimated *P *value is therefore $\frac{\Sigma \left[d^{\prime }<d\right]}{100}$. Where multiple tests are performed, a Bonferroni correction is applied by multiplying the number of random samplings by the number of tests (159 in the case of Table 3).

### Odorant binding protein paralogous groups

For this analysis, odorant binding proteins are defined as the 49 VectorBase genes annotated with InterPro domain IPR006625 (Insect pheromone/odorant binding protein PhBP). The within-species paralogues for each gene were retrieved via the Perl API from the VectorBase/Ensembl Compara database (7 species, schema version 54, August 2009). Paralogous groups (PGs) are defined as sets of genes with the same mutual paralogues. Six genes have no paralogues, two PGs contain two genes each, and one PG contains three genes. The remaining PGs contain five or more genes and are listed in Figure 4.

## Authors' contributions

RMM participated in the design of the study, its implementation, analysis and write-up. SNR and RMM participated in data preparation and web interface design. GCK coordinated the project and refined the manuscript. All authors read and approved the final manuscript.

## Supplementary Material

Additional file 1 **Figure S1. DNA/RNA/protein metabolic functions**. Pie charts for each cluster indicate the relative number of genes annotated with selected GO terms related to DNA/RNA/protein metabolism. In general, the relative location of the gene functions reflects the underlying biology. For example, DNA replication and repair are found close to each other [35], as are translation and protein transport (translocation to the endoplasmic reticulum is co-translational [36]). In contrast to previous figures, the pie chart area is not proportional to the number of genes in each cluster. The colours correspond to the following GO terms: orange, GO:0006260, DNA replication; light blue, GO:0006281, DNA repair; green, GO:0006350, transcription; yellow, GO:0006396, RNA processing; dark blue, GO:0006412, translation; vermillion, GO:0006457, protein folding; purple, GO:0015031, protein transport; black, GO:0000502, proteasome. Click here for file

Additional file 2 **Figure S2. Dynamic range of expression map input data**. Box-whisker plots indicating the maximum, minimum, upper/lower quartiles, and median for the 93 dimensions (conditions) of the normalised data used to generate the map. Some experiments exhibit a wide range of expression values, for example the embryonic developmental series, while others show a more limited range, for example the M and S form comparisons. Click here for file
