# Supplementary figures and images for: An expression map for Anopheles gambiae
Source: BMC Genomics. 2011 Dec 20;12:620. doi: 10.1186/1471-2164-12-620 (PMC3341590; doi:10.1186/1471-2164-12-620)

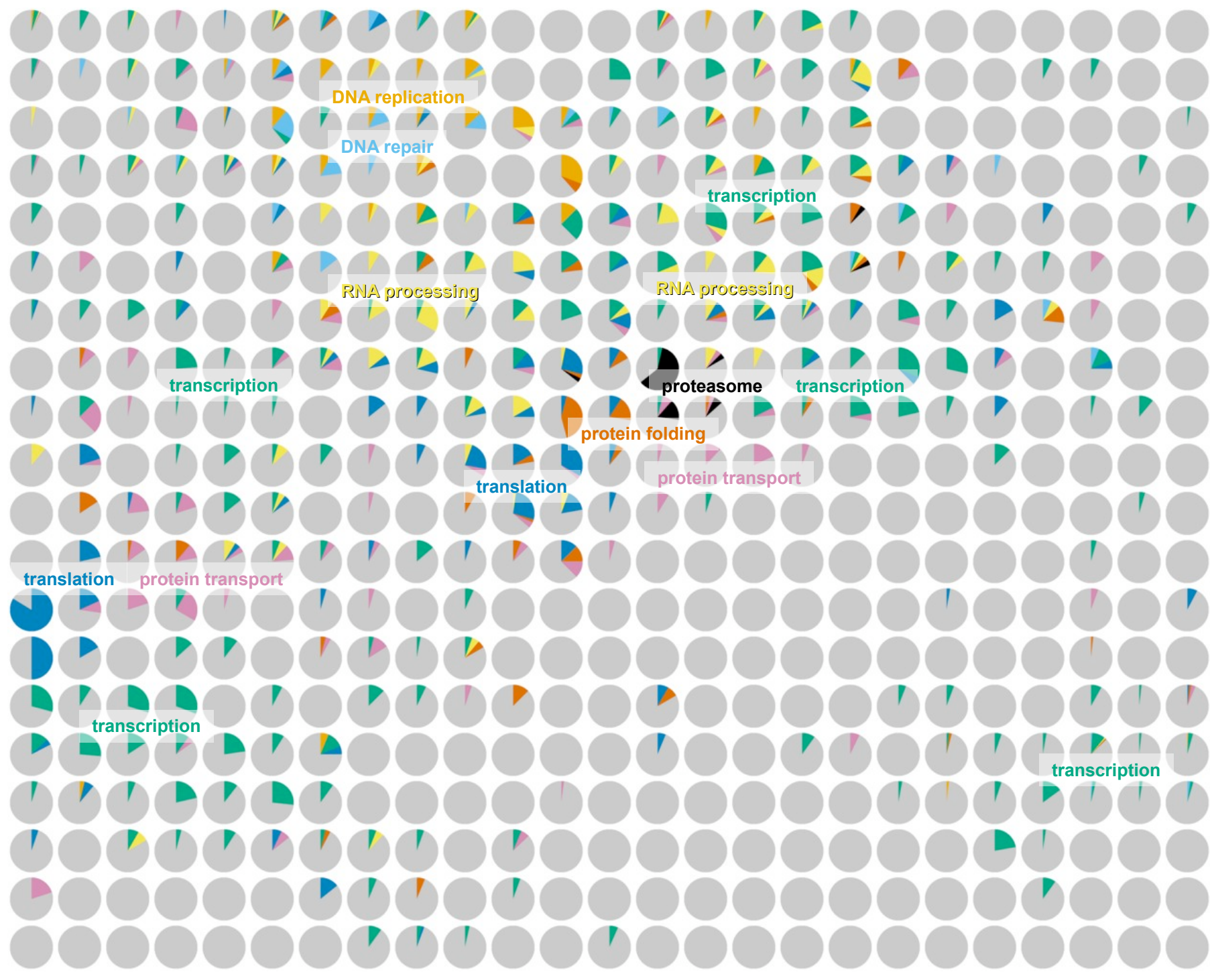

Supplement: Additional file 1 — Figure S1. DNA/RNA/protein metabolic functions. Pie charts for each cluster indicate the relative number of genes annotated with selected GO terms related to DNA/RNA/protein metabolism. In general, the relative location of the gene functions reflects the underlying biology. For example, DNA replication and repair are found close to each other [35], as are translation and protein transport (translocation to the endoplasmic reticulum is co-translational [36]). In contrast to previous figures, the pie chart area is not proportional to the number of genes in each cluster. The colours correspond to the following GO terms: orange, GO:0006260, DNA replication; light blue, GO:0006281, DNA repair; green, GO:0006350, transcription; yellow, GO:0006396, RNA processing; dark blue, GO:0006412, translation; vermillion, GO:0006457, protein folding; purple, GO:0015031, protein transport; black, GO:0000502, proteasome. [file 1471-2164-12-620-S1.PDF]

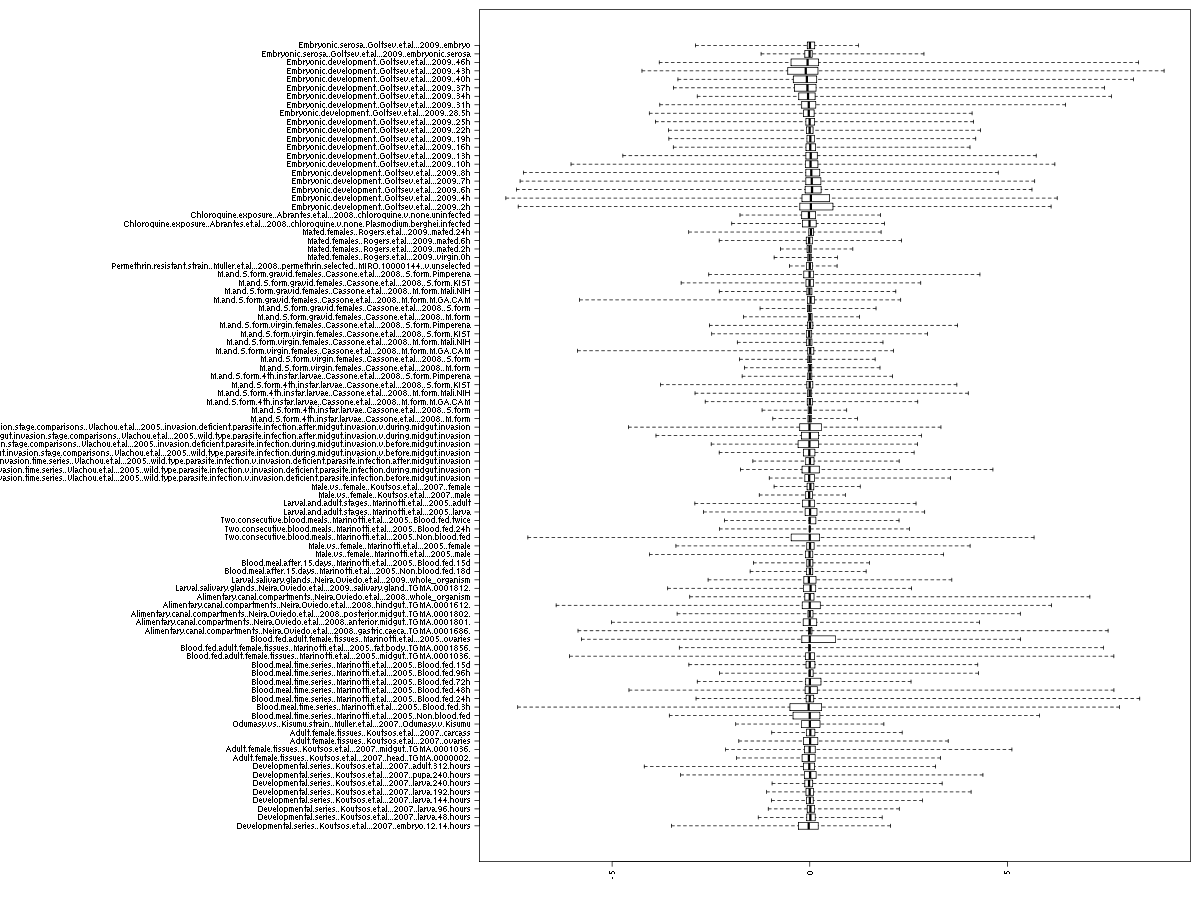

Supplement: Additional file 2 — Figure S2. Dynamic range of expression map input data. Box-whisker plots indicating the maximum, minimum, upper/lower quartiles, and median for the 93 dimensions (conditions) of the normalised data used to generate the map. Some experiments exhibit a wide range of expression values, for example the embryonic developmental series, while others show a more limited range, for example the M and S form comparisons. [file 1471-2164-12-620-S2.PNG]
